# Are Local Dairy Products Better? Using Principal Component Analysis to Investigate Consumers’ Perception towards Quality, Sustainability, and Market Availability

**DOI:** 10.3390/ani12111421

**Published:** 2022-05-31

**Authors:** Valentina Maria Merlino, Manuela Renna, Joana Nery, Arianna Muresu, Alessandro Ricci, Aristide Maggiolino, Giuseppe Celano, Barbara De Ruggieri, Martina Tarantola

**Affiliations:** 1 Department of Agricultural, Forest and Food Sciences, University of Turin, L.go P. Braccini 2, 10095 Grugliasco, TO, Italy; valentina.merlino@unito.it (V.M.M.); arianna.muresu@edu.unito.it (A.M.); 2 Department of Veterinary Sciences, University of Turin, L.go P. Braccini 2, 10095 Grugliasco, TO, Italy; joana.nery@unito.it (J.N.); alessandro.ricci@unito.it (A.R.); martina.tarantola@unito.it (M.T.); 3 Department of Veterinary Medicine, University of Bari Aldo Moro, Strada prov.le per Casamassima, Km. 3, 70010 Valenzano, BA, Italy; aristide.maggiolino@uniba.it; 4 Department of Soil, Plant and Food Sciences, University of Bari Aldo Moro, Via Giovanni Amendola, 165/a, 70126 Bari, BA, Italy; giuseppe.celano@uniba.it (G.C.); barbara.deruggieri@uniba.it (B.D.R.)

**Keywords:** consumer, milk-derived products, multivariate statistical analysis, quality, socio-demographic characteristics, sustainability, tradition

## Abstract

**Simple Summary:**

The perception of the local production concept significantly varies depending on the weight given to each defining component (e.g., quality and sustainability), which in turn depends both on the considered food product and on the consumers’ characteristics. In this research, a survey was conducted to investigate the consumer preferences for milk and cheese quality aspects and their perception of sustainability descriptors of local dairy products. In addition, the effect of socio-demographic variables on consumer preferences and attitudes definition was also explored. From the Principal Component Analysis, four main components were defined (Responsive to quality attributes, Local is better, Local is sustainable, and Availability request), combining the consumer answers about milk and cheese quality preferences and the sustainability and availability opinion. The obtained results highlight the importance of product quality aspects in the considered geographical area (South-East Italy), which is strictly linked to traditional dairy production. Higher availability and visibility of local dairy products on the market were requested by the considered consumer sample. In addition, our results showed that gender, age, place of residence, educational level, and family size significantly affected the local dairy products consumption orientation definition. These findings will support the development of more efficient and transparent communication among dairy producers and consumers in the selected market.

**Abstract:**

Consumers are increasingly aware of the benefits of local foods in terms of quality, sustainability, animal welfare, and safety. This research addresses two main questions: (i) is the perception towards sustainability aspects of local dairy products related to individuals’ preferences for milk and cheese quality aspects? (ii) are these perceptions related to people’s socio-demographic characteristics? For this purpose, a choice experiment was conducted online in Apulia (South-East Italy) involving a sample of 543 respondents. A Principal Component Analysis (PCA) was applied to analyze the consumer opinion related to dairy products’ quality attributes, sustainability, and availability on the market. From the PCA, four main components (accounting the 64.5% of the total explained variance) were defined, highlighting non-overlapping choice styles of consumers, distinguished by attitudes primarily based on quality attributes of dairy products rather than sustainability characteristics or perceived higher quality of local products. Furthermore, the Availability request component described the consumer need for higher availability and/or visibility of local dairy products on the market. The effect of gender, age, and educational status of individuals emerged as significantly important for the resulting component definitions. The obtained results clearly suggest the need to increase the efficiency of communication strategies concerning local dairy products, as well as local dairy products’ availability and visibility on the markets.

## 1. Introduction

Economic and efficiency benefits of a supply chain are known to derive from an increasingly global economy [1,2]. However, the centralization of food production results in an increased distance of food traveling and in a loss of connection between the consumers and the food they eat. The combination of these factors, in association with the ongoing COVID-19 pandemic emergency, has triggered an increased demand and expenditure for local foods [3,4,5].

The connotations of the proposed definitions for the “local food” concept ranged from distances (i.e., miles or kilometers) that the food travels from production to consumption, political boundaries and specialty criteria to more holistic approaches that also included emotional and/or ethical dimensions, such as personal relations with or within the production area (i.e., region, city, geographical zone) [6]. The absence of a unique, consistent, and globally accepted definition of the adjective “local” makes it impossible to create a standardized label for local food [7,8]. Despite the absence of specific labeling, local foods are progressively more demanded by consumers who associate the concept of local production with aspects such as freshness, high quality, greater safety, and greater socio-environmental sustainability of the resulting products [6,9,10,11]. The level of social and economic advantages reflected by local foods in relation to the local community has been addressed as a driving element for consumer choices [12]. The growing consumers, retailers, and policy interest in local food sales stimulate economic development, particularly in rural economies characterized by low incomes and outmigration [13].

The identification of consumers’ preferences underlying food values is a decisive element in improving food marketing, communication, and policymaking [14]. Several scientific studies explored the perception and awareness of consumers towards local food and their willingness to pay for traditional and territory-bounded food products [15,16,17,18,19]. Other studies investigated the association between local production and consumers’ perception of food product quality [11,20,21,22] or sustainability [23,24,25,26,27]. The effect of socio-demographic variables of individuals on shaping preferences, attitudes, and perceptions during food choice decision-making has also been highlighted [12,28,29,30,31].

However, to date, there have been few attempts to improve labeling and marketing strategies related to local food products [32,33]. As a result, further research using field experiments is needed to better understand local food messaging and the potential claims in relation to consumers’ preferences and needs in making these products more recognizable and visible [34]. In addition, to our knowledge, no studies are currently available simultaneously exploring the perception of quality and sustainability aspects and opinions on the market availability of local dairy products, particularly in geographic contexts that are traditionally and historically linked to the dairy supply chain. To fill this knowledge gap, with the present study, we aimed to assess, from a quantitative perspective, consumers’ preferences towards different quality attributes of local dairy products in relation to their perceptions of product sustainability and availability and their socio-demographic characteristics.

## 2. Materials and Methods

### 2.1. Data Collection

A choice experiment was carried out to investigate consumers’ attitudes and preferences towards local dairy products. For this purpose, a structured questionnaire was developed and sent to a sample of individuals from July to August 2020. The questionnaire items were firstly examined through a preliminary pilot survey to test the internal consistency and reliability of the developed scales using Cronbach’s formula, considering acceptable α values higher than 0.80. The reproducibility was tested using Pearson’s correlation analysis.

**Figure 1:**
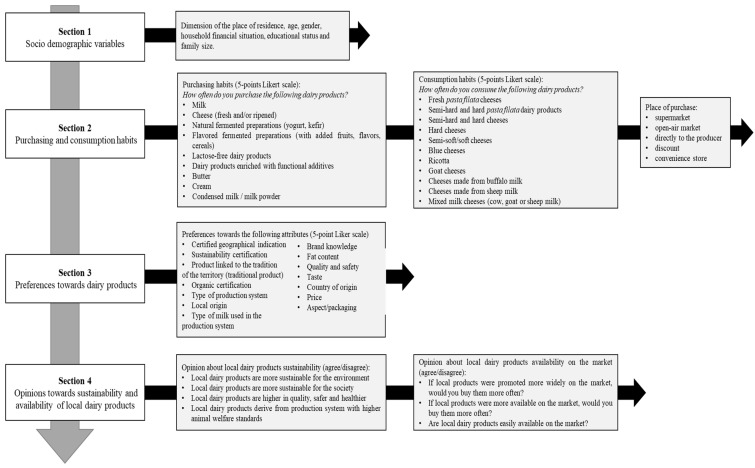
Flowchart of the questionnaire used to explore consumers’ preferences, perceptions, habits of purchasing, and consumption of local dairy products.

Due to the restrictive measures imposed by the Italian Government to limit the infection from COVID-19, the validated version of the questionnaire was submitted online only, using social media and associations of consumers to contact adult people living in the Apulia region (South-East Italy). The survey was conducted following the ethical standards set out in the Declaration of Helsinki. The questionnaire was anonymous, did not include sensitive data, and was developed in the Italian language. According to the European Innovation Scoreboard, the selected region belongs to the groups of so-called “modest and moderate” innovators [35]. The questionnaire was divided into four main sections. The flowchart of the questionnaire structure is shown in Figure 1 [36].

The first section included questions about the socio-demographic characteristics of the respondents. Information on individuals’ purchasing and consumption habits (Section 2) and preferences towards 14 milk quality attributes (Section 3) were evaluated using 5-point Likert-type scales (1 = Strongly unimportant; 2 = Unimportant; 3 = Neither important nor unimportant; 4 = Important; 5 = Strongly important) [24,37]. The selected attributes describing dairy products were chosen after an in-depth literature search to define consumers’ preferences concerning: (i) product intrinsic characteristics (type of milk used in the production system, fat content, quality and safety, taste) [38,39]; (ii) items linked to the origin (certified geographical indication, local origin, country of origin) [40,41,42,43]; (iii) extrinsic features (price, aspect/packaging, brand knowledge) [39,44,45]; and (iv) attributes describing the product environmental and social sustainability (sustainability certification, product linked to the tradition of the territory/traditional product, organic certification, type of production system) [46,47]. At the beginning of Section 3, the following three questions were included: “Do you consume dairy products?” (Answers: yes/no), “Do you consume local dairy products?” (Answers: yes/no), and “How often do you consume local dairy products?” (Answers: very often, often, sometimes, rarely, never). Finally, in Section 4, the respondents were asked their opinion (agree/disagree) about 4 attitudinal questions concerning local dairy products/production sustainability and about 3 questions concerning the availability and sourcing of local dairy products on the market. The first group of questions, related to dairy product sustainability, was included with the aim to define the consumers’ perception of the link between local production and both the environmental [48,49] and social [50] sustainability dimensions. Regarding social sustainability, product quality/safety aspects [51,52] and animal welfare [53,54] were also considered. The second group of questions, related to dairy products available on the market, was included to define the accessibility perception, availability, and visibility (referred to as right promotion) of local products on the considered marketplace, as well as the consumers’ willingness to buy more in case of an increased local products availability [55,56].

### 2.2. Data Analysis

To profile the involved consumers in terms of dairy purchasing and consumption habits, their responses to Section 2 of the questionnaire were analyzed and described qualitatively (analysis of the frequency of responses).

A Principal Component Analysis (PCA) was used to identify different consumption patterns defined by individual preferences towards the selected dairy product attributes (Section 3) and the perception of local products’ sustainability and availability on the market (Section 4) [57]. The PCA is a technique for data simplification used in statistics with the aim of reducing the number of variables describing a multivariate complex dataset to a smaller number of latent variables, limiting the loss of information as much as possible and enabling the interpretation of the starting data matrix [58]. In particular, this technique allows the information provided by responses to a large number of questions to be condensed into a small number of orthogonal components [59]. This multivariate statistical methodology is widely used in market research, and particularly in consumer studies, to assess individuals’ preferences and attitudes based on purchasing behavior and product characteristics [28,57,59,60]. This approach has proven useful in making critical decisions applicable to the real world of the agri-food sector [61,62].

In our study, the PCA was applied to the means of preference scores given to the dairy product attributes (preferences questions in Section 3 of the questionnaire) and to the responses given to Section 4 (sustainability and availability perception questions); in the latter case using the codified answers (Yes = 1/No = 0) [63]. A total of 21 questions regarding perceptions and preferences found about dairy products were aggregated into a minor number of orthogonal principal components (PCs).

The choice to use both continuous (Section 3) and binary (Section 4) variables in the model [61,63] was helpful in defining consumption patterns in which both preferences towards product attributes and individuals’ attitudes, beliefs, and perceptions about local products sustainability coexist. Despite conflicting theories, several studies have demonstrated the usefulness of using binary variables in PCA when results have direct implications in the real world [61,63,64].

From the correlation matrix of the consumers’ responses, the PCA calculated the eigenvalues to allow for the analysis of the answers. This approach allowed to produce the weights that convert the original variables into a reduced set of new representative variables identified by the component scores (loadings) [65]. These scores indicate the influence level of data in the new factors. In this study, a Varimax rotation was used to create an orthogonal solution for the formation of the weights, in which the new variable with the highest explained variance is projected onto the first axis, and the new variable, second in variance size, is projected onto the second axis, and so on. The rotated component matrix analysis allowed us to assess how much and whether questions designed to measure a particular component contribute to the component itself [30,66].

In our study, the influence of a given variable in the component definition was rejected for a value below 0.30, it was accepted with ratios between 0.35 and 0.7, and it was considered to have a strong influence if it had a ratio above 0.7 [67].

To measure how well the provided data were suitable for PCA and to test the null hypothesis (the variables are orthogonal, i.e., not correlated), the Kaiser–Meyer–Olkin (KMO) and Bartlett’s sphericity tests were used, respectively. The KMO test provides a 0 to 1 index and considers the correlation coefficients to indicate the sample suitability. If the KMO test returns a value between 0.8 and 1 (high correlation), sampling is considered adequate; if the returned value is near 0, the test suggests the inadequacy of the dataset for the PCA, while values below 0.5 indicate that sampling is not adequate [68]. Bartlett’s sphericity test was performed to verify that a variable reduction technique, such as the PCA, could compress the data significantly. Specifically, it relies on comparing two matrices (of observed correlation with identity matrix) and testing whether there is some redundancy between variables that can be summarized with a reduced number of factors. If the two considered matrices differ significantly, the data reduction technique is appropriate. Then with a *p*-value of the test below the significance level (*p*-value < 0.05), the data set is suitable for the chosen reduction technique [69].

Finally, to test whether there were significant differences between the groups identifying different socio-demographic variables in the PCA components, two non-parametric tests based on differences between the mean ranks were used. The Mann–Whitney U test was used to compare the gender-related differences (two categorical groups: males and females). The Kruskal–Wallis H test was instead used in the case of more categorical independent groups, such as for age, place of residence, educational level, household financial status, and family size [70]. All these variables were used as grouping parameters together with the loadings extracted for the obtained new components by the PCA. In the case of significant differences that emerged from Kruskal–Wallis multiple comparisons, the Mann–Whitney test also was used to describe which groups were significantly different for each factor. All the analyses were performed using the IBM SPSS Statistics software, Version 25.0 for Windows (IBM Corp., Armonk, NY, USA).

## 3. Results and Discussion

### 3.1. Consumers’ Profile

A total of 543 individuals participated in this study, filling out the questionnaire and resending it to us by email. The socio-demographic characteristics of the sample involved in the study are presented in Table 1.

The sample was balanced in terms of gender. The interviewed individuals were proportionally distributed in the different age groups, except for the over 65, who were less represented than the other age groups. They were mainly university graduates with a satisfactory average annual household income. The sample is relatively well distributed in terms of living location: the involved consumers lived in rural areas (36%), in medium-large cities (33%), while the 22% of them lived in cities with more than 250 thousand inhabitants. Compared to the population of Apulia on 1 January 2020 (3,933,777 total inhabitants, Apulia being the eighth region in Italy in terms of the number of inhabitants), the distribution between men and women in the sample involved is comparable to that of the region of origin (49% men and 51% women), as is the distribution in the different age groups. In fact, on 1 January 2020, the population of Apulia was distributed as follows: 12% individuals between 18 and 25 years old, 15% between 26 and 35 years old, 17% between 36 and 45 years old, 21% between 46 and 55 years old, 19% between 56 and 65 years old and 10% of over 65s (up to 80 years old, corresponding to the oldest respondent to our survey). In the same period, 1,595,981 families lived in Apulia, 0.6% more than the previous year (+0.5% of the national figure). The average number of members per household was 2.5, slightly above the national average of 2.3. In 2020, 17% of the total population had a primary school license, 32% a lower secondary school license, and 32% an upper secondary school license, while the incidence of university degrees increased compared to the previous year by +6.6% for a total of 19% [71].

It is, therefore, affirmable that the sample involved largely represents the population of origin with some differences probably due to the online data collection method, which, as usual, makes it difficult to collect a representative sample due to the demographic characteristics of the web users.

### 3.2. Purchasing and Consumption Habits of Dairy Products

Ninety-seven percent of the total number of respondents stated that they consume dairy products, of which 96% were locally produced. Twenty-three percent of the respondents said they consume local dairy products very often, 49% often, 20% sometimes, while only 7% rarely and 1% never. This result highlights an evident propensity on the part of the sample involved to choose a traditional product (identified in dairy products) and a local one. The correlation between the two components (tradition-local) in the decision-making process of choice could define a model of choice typical of the area under study, which, as previously mentioned, is characterized by a deep-rooted productive tradition. Probably, these consumers recognize in these products the cultural importance of the territory of origin, correlating to this product a process of habitual choice directed almost exclusively to products of local tradition [72]. The latter assumption is in line with other studies in which consumers’ consideration of local food emerged to better satisfy their habits and needs [73,74]. This sentiment manifests their togetherness and their sense of being connected to the place where they live. Many researchers have already noted such a phenomenon: when buying directly from the local farmers, consumers especially emphasize the importance of supporting local producers [75], but also pride in their region as well as product freshness [76]. Buying local food is a means of building community, of gaining a sense of belonging and self-identity [77]. This phenomenon of sentimental bonding has been described as *topophilia* [78] and later as *terraphilia* [79].

**Figure 2:**
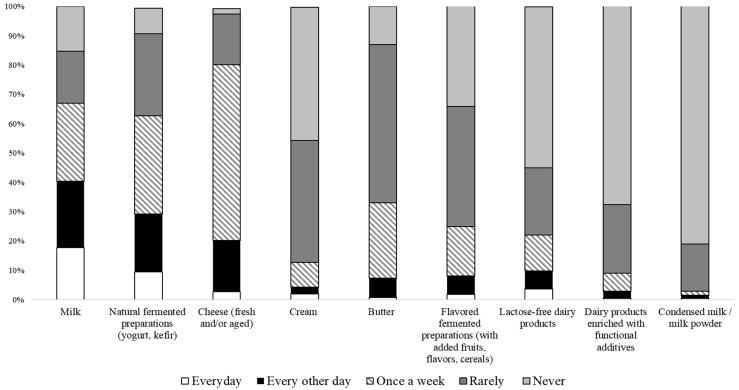
Frequency of purchase of different dairy products.

The frequency of purchase declared by the involved consumers for the different considered dairy products is reported in Figure 2.

**Figure 3:**
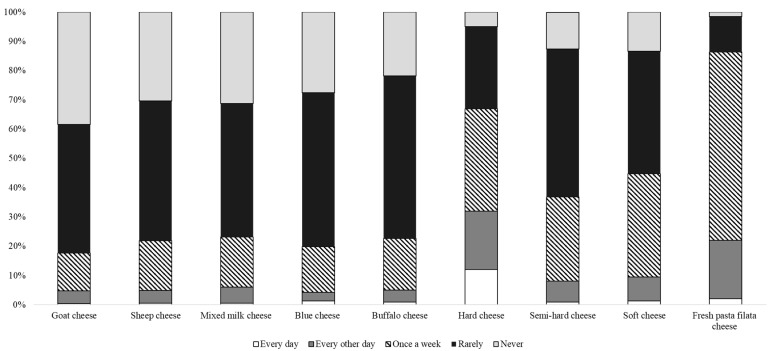
Frequency of consumption of different cheeses.

About 20% and 10% of the interviewed consumers bought milk and yogurt on a daily basis, respectively. In general, these two products were the most frequently purchased ones, followed by cheese. In contrast, cream and condensed milk were the least purchased products by the surveyed consumers. This is in accordance with the purchasing choice of Italian consumers [80,81]. In general, about half of the sample consumed cheese every other day or weekly, particularly fresh, hard, and semi-soft cheeses. Cheeses made using cow milk were the most preferred by consumers, while those made using milk from other ruminant species (goats, sheep, and buffaloes) were consumed less frequently (Figure 3).

If the choice of cow’s milk products was in line with the choices of Italian consumers, the choice of hard and semi-hard cheeses is in contrast with the national consumer sample who, in the last years, were more and more oriented to the purchase and consumption of fresh products [80]. This result highlights once again how tradition influences the choices of individuals who are loyal to a product culturally integrated into their diet based on family habits [82].

Our results showed that, as the preferred place to purchase dairy products, the respondents indicated supermarkets (49%), followed by producers (direct sales) (26%), convenience stores (15%), outdoor markets (6%), and discount stores (5%). The choice of the producer as a point of purchase for dairy products highlights consumers’ search for safety, quality, and trust, which can only be found in a well-known and habitual place of purchase where the company image is a guarantee of quality [13,83]. However, large-scale retail trade remains the place of choice for the purchase of products, which presupposes how retailers have developed effective tools to reduce the distance between the primary producer and final consumers. It is perceived that successful communication with the final consumer has helped to develop mutual trust and differentiate local products from other conventional and non-local products [55,84,85,86].

### 3.3. Dairy Products Consumption Patterns Based on Quality, Sustainability, and Availability Perception of Individuals

Based on the consumers’ responses to the questions about the preferences of the selected quality attributes and the perception of dairy product sustainability and availability, four components were identified in the PCA, with a total explained variance equal to 64.5%. The rotated component matrix and the questions used to generate the component scores are presented in Table 2.

The first four PCs generated from the analysis were retained in accordance with the Kaiser criterion (eigenvalue > 1) [87] to represent the variations meaningfully in perceptions about local and sustainability on the one hand and the individuals’ revealed preferences regarding dairy products. The Varimax rotated factor loadings with an absolute value greater than 0.3 represent a strong influence on the pattern definition (Table 2).

The first PC (36.21% of the total explained variance) is associated with a dietary pattern characterized by relatively high loadings on all the statements related to the preferences determination about the quality attributes of dairy products. This component (PC1), named “Responsive to quality attributes”, identified consumers’ choices oriented towards a high degree of attention over the intrinsic and extrinsic aspects of the products. All the drivers that affect the consumer decision-making process during purchase emerged as important in this component, excluding price and taste. Price is very commonly identified as an influential factor in consumer attitudes; therefore, our findings can be explained in the kind of sample that showed a satisfactory financial situation [88,89]. Moreover, other studies showed that usually evaluated factors, such as price and organoleptic characteristics, are not considered very important during choice because they are considered pre-requisites already established by the consumer. This was found, for example, in a study on meat consumers where it was found that individuals who were regular purchasers of traditionally (and locally) branded meat rated organoleptic attributes as unimportant [72].

The PC2 (11.38% of the total explained variance) represented covariation between a high level of perceived benefits of local products linked to a higher quality of the product. This PC2 revealed consumption preferences oriented to the local origin, the tradition, and the relationship between the production process and the territory, probably linked to higher standard quality and safer and healthier production. In this component, named “Local is better”, a clear correlation between the local production and higher perceived product quality, as linked to the tradition and the territory, emerged. These findings are consistent with previous studies [3,90].

The PC3 (9.92% of the total explained variance) had positive and significant loadings on questions related to the perception of local product sustainability only. This PC, named “Local is sustainable”, highlighted an attitude concerning the link between the local production and the environmental and social dimensions of sustainability. These are in line with the findings of previous studies that found only positive attitudes toward local foods among consumers. Participants associated local food with supporting the local economy and environmental benefits [91]. However, there is evidence that the local food is not always more sustainable (Stein and Santini 2021). Some researchers suggested how the carbon footprint from some local foods, assessed by life cycle assessment, is higher than the longer chain alternative because local foods do not benefit from the scale economies of mass production and transportation [3]. However, the right management of the resources of the territory and of the local ecosystems can represent an adequate instrument of valorization of the autochthonous elements of the territory and of the products deriving from them the safeguard of the surrounding environment [49]. In addition, this PC was characterized by a high degree of consensus about the positive relationship between local products and both animal welfare and higher product quality. Local food was expected to be produced in small-scale systems and was acknowledged to use sustainable local resources and preserve biodiversity. Small-scale and extensive systems are perceived as inherently welfare friendly. Animal welfare is a credence quality attribute [92,93,94] that is of great interest to consumers [95], often associated with the greater ethicality of dairy production systems located in marginal areas [96].

The last PC (6.99% of the total explained variance), named “Availability request” (PC4), was mainly defined by the opinion that local products are not largely available on the market, creating a state of general dissatisfaction by part of the respondents. The latter condition could be inferred from the respondents’ expressed willingness to buy more local products if they were more available and better promoted in the market.

The European Union (EU) has granted legal protection for the names of food products closely identified with where they were produced or methods used in production through the European Union Protected Designation of Origin (PDO) or Protected Geographical Designation (PGI) certifications. Research indicates that these EU protections are recognized by consumers and can add value to food products [97]. This allows consumers to make more informed purchasing choices. On the contrary, the lack of clarity in the definition of local production, as well as poor communication of the origin of products, can lead to difficulties in the decision-making process by consumers. For example, Pirog [11] found that eco-labels, or eco-sustainable labels, can be an effective and receptive means of informing consumers about the environmental performance of the products or production systems from which they come and can also inform consumers about measures taken by producers to minimize the environmental impact of the product.

### 3.4. Effect of Socio-Demographic Variables on Local Dairy Products Sustainability and Quality Perception

The results of the Mann–Whitney U tests for the assessment of gender differences in the components’ characteristics are reported in Table 3.

No significant differences were found between genders in the perception and preferences profiles towards dairy products defined by the components “Responsive to quality attributes”, “Local is sustainable”, and “Availability request”. On the contrary, women were more likely to consider the benefits of local production, as expressed in the “Local is better” component when compared to men.

Gracia et al. [12] revealed that social influence does indeed affect willingness-to-pay values, but the effects are different between men and women. Women appear to have more positive attitudes towards local foods than men; therefore, they may be more willing to purchase and pay for local foods. In addition, our results highlight a greater propensity of the female gender to choose local products due to the search for a safer, familiar, and higher quality product [65,98], emphasizing the anthropic view [99,100] of the concept of locally found in household purchasing managers [101].

On the other hand, the age of the respondents seemed to significantly influence the consumers’ perceptions and preferences expressed within all the obtained PCs (Table 4).

In fact, in the two PCs, “Responsive to quality attributes” and “Local is sustainable” (PC1 and PC3), young consumers (under 25) showed less sensitivity to the issue, which is instead felt more in the older age groups. On the contrary, the link between local production and product quality, expressed in the “Local is better” PC, was more appreciated by the young consumers (18–25 y) who, at the same time, seemed to show a higher demand for greater availability of local products on the market. This result highlights the greater sensitivity of more mature consumers to the dimensions of sustainability that characterize local products. On the contrary, more concrete aspects of product quality, intrinsic and extrinsic attributes, seem to be decisive in the choices of young consumers who expressed, at the same time, the lack of product availability. These results are in contrast with published literature, which often portrays young people as more sensitive to problems linked to sustainability and environmental protection [31,102,103]. Probably, the experience that characterizes more mature consumers, in the analyzed context, makes them confident about the quality and availability of the product, emphasizing the aspects of credence of dairy products linked to the territory. The product availability problem, in addition, may also be attributable to a lack of communication of local origin information on the label, which, as emerges in the literature, is often consulted by young consumers to access information. Moreover, the main places of local product sales, represented by company outlets or open-air markets, are probably not the places mainly chosen for shopping by the younger generations [104,105,106].

Significant differences in terms of respondents’ place of residence dimension emerged in the definition of the “Availability request” PC. Since the availability of local and sustainable foods is beginning to spread to mainstream retail stores, it seems that the problem of low availability and recognisability of local products on the market is felt more strongly in urban areas [107] (Table 5).

Retailer strategies for locally produced foods are complicated by the fact that these items can compete with retailers’ private brands and the national brands they carry. As a result, locally produced foods, private brands, and national brands compete for the same shelf space at the retail level [97]. To that end, proper and strategic communication of product and local brand value can create differentiation in the marketplace as well as support the added value of local products. Large retail distribution is seeking methods to communicate to consumers that they have locally produced foods in their stores, and state-sponsored designations could potentially be an important method for it to use [85,108].

Significant differences in terms of respondents’ educational status emerged in the definition of the “Local is better” PC (Table 6).

Higher levels of education appear to correlate with greater recognition of the intrinsic quality of local products. The financial condition seems not to influence the considered consumption patterns described in all the PCs (Table 7).

This result is also confirmed by the market studies carried out by the National Institute of Services for the Agricultural Food Market (ISMEA) on the consumption patterns of dairy products, which emerged how the income of families is not influenced by the definition of purchase preferences and the evolution of consumption patterns that have characterized the dairy sector in Italy in recent years [80,81,109].

Finally, the analysis of the effect of family size and, consequentially, the presence of children (up to 2) in the household revealed no significant differences in the PCs’ definition (Table 8).

This result is in countertendency with other studies on consumer preferences for food products of animal origin in which greater attention was found to aspects of food quality and safety in families with children [101,110]. Annunziata et al. [111] highlighted how family composition and, in particular, the presence and the age of children affect food choices regarding sustainability and local origin. Probably, the presence of children in the 73% of the population sampled in our study defines a standard choice orientation attributable to the entire sample. Therefore, given the average levels of preference, we can confirm that the presence of children determines positive attitudes in the evaluation of local production related to sustainability and higher product quality. In the case of the component related to the issue of product availability, did purchasing managers in larger households provide more attention. Probably the scarce availability perceived by these individuals, associated with a greater need for product service, stockability and price/quality ratio, induces these consumers to make choices towards more widely available products at more competitive prices.

## 4. Conclusions

This study shows how the attitudes, perceptions, and preferences of consumers towards local production are strictly influenced by traditions rooted in the territory, habits and familiarity with the product, and by the socio-demographic characteristics of individuals. Firstly, in the research area, where the dairy tradition is ancient and felt by the population, the purchasing habits of milk-based products reflect feelings of identity, culture, values, and sense of belonging to the territory that consumers arise in the decisions of choice. Furthermore, from the analysis of the main PCs, within the same survey sample, different consumption profiles emerged oriented towards the evaluation of the most tangible aspects of the local products, but also to identifiable beliefs in the safety and sustainability of local products. Finally, the differences that emerged in the definition of consumption patterns as a function of socio-demographic characteristics highlight the importance of targeting productions and the development of ad hoc communication campaigns for different consumer profiles. Among the limitations of this research, there is the consideration of the limited area, which, however, could be a starting point for the development of new studies that can be extended to other Italian and European regions. Our study contributes not only to the enrichment of literature concerning the study of consumer perception towards local productions but also provides a useful tool for supply chain actors to increase the efficiency of the communication strategies, as well as product availability/visibility on the markets. In fact, the opinion of the involved consumers regarding the poor visibility of local products on the market could be used as a starting point by companies to develop communication campaigns and planning strategies for product label improvement. In particular, the use of impactful keywords and claims could make the products more visible and understandable to potential buyers.

## Figures and Tables

**Table 1:** Socio-demographic characteristics of the participants.

Variable	Description	Frequency	Share of Sample (%)
Gender	Female	299	56
	Male	239	44
Age (years)	18–25	68	12
	26–35	146	27
	36–45	99	18
	46–55	136	25
	56–65	73	13
	>65	24	4
Education	Lower secondary school	14	3
	Upper secondary school	172	32
	Degree or higher	354	66
Household financial situation	Very satisfactory	25	5
	Satisfactory	316	59
	Sufficient to make ends meet	175	32
	Difficult	18	3
	Very difficult	6	1
Residence	Town with more than 500,000 inhabitants	34	6
	Cities with 250–500,000 inhabitants	87	16
	Cities with 100–250,000 inhabitants	47	9
	Towns with 50–100,000 inhabitants	109	20
	Towns with 50,000 inhabitants	71	13
	Country-rural location	194	36

**Table 2:** Varimax rotated PCA: dairy products sustainability and quality components (*n* = 543). Each component is named in accordance with the consumption models described by the significance of values.

Variables	Principal Components			
	PC1 (Responsive to Quality Attributes)	PC2 (Local Is Better)	PC3 (Local Is Sustainable)	PC4 (Availability Request)
**Preferences Questions (Degree of Preferences for Quality Attributes of Dairy Products** **)**				
Certified geographical indication	0.840			
Sustainability certification	0.815			
Product linked to the tradition of the territory	0.789	0.324		
Organic certification	0.779			
Type of production system	0.735			
Local origin	0.735	0.398		
Type of milk used in the production system	0.730	0.363		
Brand knowledge	0.720			
Fat content	0.684			
Quality and safety of the production	0.376	0.801		
Taste		0.776		
Country of origin	0.494	0.631		
Price		0.352		
Aspect/packaging	0.372			
**Sustainability Perception Questions**				
Local dairy products are more sustainable for the environment			0.767	
Local dairy products are more sustainable for the society			0.722	
Local dairy products are better in quality, safer, and healthier		0.325	0.666	
Local dairy products derive from the production system with higher animal welfare standards			0.612	
**Local Products Availability**				
If local products were promoted more widely on the market, would you buy them more often?				0.832
If local products were more available on the market, would you buy them more often?				0.816
Are local dairy products easily available on the market?				−0.484

Kaiser–Meyer–Olkin index = 0.90. Bartlett’s sphericity test: Chi square = 5481.042; *p*-value = 0.000. Non-significant values (<±0.3) are not shown.

**Table 3:** Effects of gender on the principal components’ definition.

Principal Component	*n*	Mean	SD	Mean Rank		Mann–Whitney U	*p*-Value
				Man	Woman		
PC1-Responsive to quality attributes	543	0.001	0.999	261.15	276.22	33,757.000	0.264
PC2-Local is better	543	−0.001	0.999	259.61	277.47	33,386.000	0.000
PC3-Local is sustainable	543	−0.004	1.003	270.55	268.65	35,507.000	0.888
PC4-Availability request	543	−0.003	1.002	269.72	269.32	35,707.000	0.976

*n*: number of samples; SD: standard deviation.

**Table 4:** Effect of age on the principal components’ definition.

Principal Component	Age Range *	*n*	Mean Rank	Kruskal–Wallis H	*p*-Value
PC1-Responsive to quality attributes	18–25 ^a^	68	216.11	20.346	0.000
	26–35 ^a,b^	146	254.96		
	36–45 ^b^	99	268.96		
	46–55 ^c^	133	311.65		
	56–65 ^b,c^	73	293.41		
	>65 ^a,b,c^	24	261.73		
	Total	543			
PC2-Local is better	18–25 ^a^	68	329.14	34.411	0.006
	26–35 ^a^	146	297.90		
	36–45 ^a^	99	293.27		
	46–55 ^b^	133	236.03		
	56–65 ^b^	73	234.05		
	>65 ^b^	24	179.56		
	Total	543			
PC3-Local is sustainable	18–25 ^a^	68	233.98	14.318	0.003
	26–35 ^a,b^	146	247.12		
	36–45 ^c^	99	290.54		
	46–55 ^b,c^	133	282.76		
	56–65 ^c^	73	311.55		
	>65 ^a,c^	24	274.69		
	Total	543			
PC4-Availability request	18–25 ^a^	68	326.40	16.663	0.007
	26–35 ^b^	146	280.72		
	36–45 ^a,b^	99	279.06		
	46–55 ^b,c^	133	258.21		
	56–65 ^c^	73	225.14		
	>65 ^a,b,c^	24	254.60		
	Total	543			

* For each component, age groups with the same superscript letter (^a,b,c^) are not significantly different (α = 0.05, Mann–Whitney test, pairwise comparison).

**Table 5:** Effect of place of residence dimension on the principal components’ definition.

Principal Component	Place of Residence *	*n*	Mean Rank	Kruskal–Wallis H	*p*-Value
PC1-Responsive to quality attributes	Rural location	194	289.13	6.305	0.278
	Towns with 50,000 inhabitants	70	255.80		
	Towns with 50–100,000 inhabitants	109	271.70		
	Cities with 100–250,000 inhabitants	45	267.93		
	Cities with 250–500,000 inhabitants	87	249.37		
	Towns with more than 500,000 inhabitants	34	240.16		
	Total	539			
PC2-Local is better	Country-rural location	194	267.51	3.354	0.646
	Towns with 50,000 inhabitants	70	263.14		
	Towns with 50–100,000 inhabitants	109	267.36		
	Cities with 100–250,000 inhabitants	45	288.67		
	Cities with 250–500,000 inhabitants	87	287.21		
	Towns with more than 500,000 inhabitants	34	238.07		
	Total	539			
PC3-Local is sustainable	Country-rural location	194	279.96	3.505	0.623
	Towns with 50,000 inhabitants	70	264.24		
	Towns with 50–100,000 inhabitants	109	252.31		
	Cities with 100–250,000 inhabitants	45	265.22		
	Cities with 250–500,000 inhabitants	87	284.09		
	Towns with more than 500,000 inhabitants	34	251.99		
	Total	539			
PC4-Availability request	Country-rural location ^a^	194	270.55	7.368	0.035
	Towns with 50,000 inhabitants ^a^	70	273.31		
	Towns with 50–100,000 inhabitants ^a^	109	264.88		
	Cities with 100–250,000 inhabitants ^a^	45	266.13		
	Cities with 250–500,000 inhabitants ^a^	87	249.61		
	Towns with more than 500,000 inhabitants ^b^	34	333.75		
	Total	539			

* For each component, places of residence with the same superscript letter (^a,b^) are not significantly different (α = 0.05, Mann–Whitney test, pairwise comparison).

**Table 6:** Effect of educational status on the principal components’ definition.

Principal Component	Educational Level *	*n*	Mean Rank	Kruskal–Wallis H	*p*-Value
PC1-Responsive to quality attributes	Lower secondary school	14	320.43	1.585	0.453
	Upper secondary school	171	266.87		
	Degree or higher	352	267.99		
	Total	537			
PC2-Local is better	Lower secondary school ^a^	14	198.00	13.536	0.001
	Upper secondary school ^a^	171	239.32		
	Degree or higher ^b^	352	286.24		
	Total	537			
PC3-Local is sustainable	Lower secondary school	14	278.4	0.11	0.946
	Upper secondary school	171	271.1		
	Degree or higher	352	267.6		
	Total	537			
PC4-Availability request	Lower secondary school	14	210.79	2.861	0.239
	Upper secondary school	171	279.46		
	Degree or higher	352	266.23		
	Total	537			

* For each component, education levels with the same superscript letter (^a,b^) are not significantly different (α = 0.05, Mann–Whitney test, pairwise comparison).

**Table 7:** Effect of household financial situation on the principal components’ definition.

Principal Components Household Financial Situation		*n*	Mean Rank	Kruskal–Wallis H	*p*-Value
PC1- Responsive to quality attributes	Very difficult	6	236.33	5.256	0.262
	Difficult	18	342.53		
	Sufficient to make ends meet	174	270.12		
	Satisfactory	314	263.07		
	Very satisfactory	25	290.52		
	Total	537			
PC2- Local is better	Very difficult	6	237.67	4.150	0.386
	Difficult	18	223.53		
	Sufficient to make ends meet	174	279.31		
	Satisfactory	314	269.60		
	Very satisfactory	25	229.92		
	Total	537			
PC3- Local is sustainable	Very difficult	6	212	4.195	0.380
	Difficult	18	311		
	Sufficient to make ends meet	174	255		
	Satisfactory	314	275		
	Very satisfactory	25	277		
	Total	537			
PC4- Availability request	Very difficult	6	143.00	5.951	0.203
	Difficult	18	245.14		
	Sufficient to make ends meet	174	268.31		
	Satisfactory	314	270.08		
	Very satisfactory	25	307.64		
	Total	537			

**Table 8:** Effect of family size on the principal components’ definition.

Principal Component	Family Size	*n*	Mean Rank	Kruskal–Wallis H	*p*-Value
PC1-Responsive to quality attributes	1 component	51	254.45	5.232	0.207
	2 components	97	262.99		
	3 components	107	277.87		
	4 components	222	280.26		
	5 or more components	60	233.62		
	Total	537			
PC2-Local is better	1 component	51	247.69	5.515	0.210
	2 components	97	281.06		
	3 components	107	278.76		
	4 components	222	256.20		
	5 or more components	60	241.57		
	Total	537			
PC3-Local is sustainable	1 component	51	244.9	4.637	0.346
	2 components	97	278.5		
	3 components	107	276.8		
	4 components	222	275.1		
	5 or more components	60	237.8		
	Total	537			
PC4-Availability request	1 component	51	278.78	2.352	0.671
	2 components	97	266.81		
	3 components	107	256.19		
	4 components	222	267.52		
	5 or more components	60	292.53		
	Total	537			

## Data Availability

The data presented in this study are available on request from the corresponding author.
